# Circular RNA TLK1 Exerts Oncogenic Functions in Hepatocellular Carcinoma by Acting as a ceRNA of miR-138-5p

**DOI:** 10.1155/2022/2415836

**Published:** 2022-03-22

**Authors:** Yan Lu, Yao Liu, Ke Zhang, Li Jiang

**Affiliations:** ^1^Department of Hepatobiliary Surgery, Beijing Ditan Hospital Capital Medical University, 100015 Beijing, China; ^2^Department of Surgery, Fuwai Hospital, Chinese Academy of Medical Science, 100037 Beijing, China

## Abstract

Mounting evidence has shown that circular RNAs (circRNAs) function as key regulators in carcinogenesis and cancer progression, and this study is aimed at investigating the regulatory functions of circRNA TLK1 (circ-TLK1) in hepatocellular carcinoma (HCC). We observed that circ-TLK1 was highly expressed in HCC samples, and its high expression was closely associated with poor clinicopathological variables of HCC patients. The results of functional experiments revealed that knockdown of circ-TLK1 remarkably inhibited the proliferation, migration, invasion, and EMT of HCC cells, while circ-TLK1 overexpression promoted these malignant behaviors. Moreover, we noted that circ-TLK1 was capable of binding to miR-138-5p and upregulating its target gene, SOX4 in HCC. Based on rescue assays, miR-138-5p inhibition partially suppressed the effects of circ-TLK1 knockdown on the malignant behaviors of HCC cells. In short, this study is the first to indicate that circ-TLK1 functions as an oncogene in HCC progression partly through acting as a ceRNA of miR-138-5p, which may be a promising target for HCC therapy.

## 1. Introduction

Hepatocellular carcinoma (HCC) accounts for 90% of cases of primary liver cancer and is one of the leading causes of cancer-related deaths worldwide. In spite of significant advancement in HCC management, its incidence continues to rise [1]. At present, surgical resection is the primary therapeutic method for HCC, but owing to the high rate of metastasis and postoperative recurrence, the overall prognosis of HCC patients remains unfavorable [2, 3]. Therefore, a better understanding of the molecular mechanisms underlying HCC invasion and metastasis may provide a new idea for the treatment of HCC.

Circular RNAs (circRNAs) are a recently discovered subclass of noncoding RNA transcripts characterized by covalently closed continuous loops with neither 5′ caps nor 3′ poly (A) tails [4]. circRNAs are more stable and conservative than linear RNAs and abounds in various organisms. Emerging evidence indicates that circRNAs serve important regulatory roles in many human diseases, particularly cancers. They have become a new research hotspot in the field of cancer biology [5, 6]. circ-TLK1 (circbase ID: hsa_circ_0004442), derived from backsplicing the TLK1 mRNA, plays an oncogenic role in renal cell carcinoma [7]. Besides, inhibition of circ-TLK1 suppresses the progression of glioma [8]. In this study, we aimed to investigate how circ-TLK1 affects HCC progression as well as the potential regulatory mechanisms it holds.

## 2. Materials and Methods

### 2.1. Clinical Tissue Samples

87 pairs of HCC tissues and adjacent nontumor tissues were gathered from patients who underwent surgery from 2018 to 2020 at hospital. These patients experienced no systemic chemotherapy or radiotherapy before surgery. After collection, all tissue samples were immediately snap-frozen in liquid nitrogen and stored at −80°C. This study was approved by the Ethical Committee of Hospital, and written informed consent was obtained from all patients.

### 2.2. Cell Lines

Four HCC cell lines, HepG2, SK-hep1, HCCLM3, Huh7, and one normal fetal hepatocyte cell line, LO2, were cultured in Dulbecco's modified Eagle's medium (DMEM; HyClone, Logan, UT, USA) containing 10% fetal bovine serum (FBS; HyClone), 100 U/ml penicillin, and 100 mg/ml streptomycin at a condition of 5% CO_2_ and 37°C.

### 2.3. Cell Transfection

To construct circ-TLK1 overexpression plasmid, the full-length circ-TLK1 cDNA was synthesized and cloned into the pcD-ciR vector (Geenseed Biotech Inc., Guangzhou, China). The empty vector was used as negative control. The small interfering RNA (siRNA) of circ-TLK1 (si-circTLK1), miR-138-5p mimics/inhibitor, and their negative controls were purchased from Guangzhou RiboBio Co., Ltd. (Guangzhou, China). Cells were seeded in 6-well plates and cultured to 60–70% confluence. Lipofectamine 2000 (Invitrogen, Carlsbad, CA, USA) was used for cell transfection.

### 2.4. RT-qPCR Analysis

Total RNA was extracted using TRIzol reagent (Invitrogen) from tissues and cells, and cDNA was then synthesized using the PrimeScript RT reagent kit (TaKaRa, Dalian, China). Thereafter, qPCR reactions were carried out using a SYBR Green PCR Kit (TaKaRa) on an iCycleriQ™ Real-Time PCR Detection System (Bio-Rad Laboratories, Hercules, CA, USA). Relative gene expression was calculated using the 2^−*ΔΔ*Ct^ method [9]. GAPDH or U6 was used as internal controls.

### 2.5. MTT Assay

Cells were plated into 96-well plates at a density of 2 × 10^3^ cells/well. At the indicated time, 20 *μ*l of MTT solution (5 mg/l; Sigma-Aldrich, St. Louis, MO, USA) was added to each well, and the plates were incubated for another 4 h. After replacing the medium with 100 *μ*l of DMSO, the optical density value was measured by a microplate reader (Multiskan EX, Lab Systems, Helsinki, Finland) at 570 nm.

### 2.6. Transwell Assay

Cells were suspended in serum-free medium at a density of 5 × 10^5^ cells/ml. 200 *μ*l of cell suspension was plated into the upper chamber of Matrigel-uncoated or -coated transwell inserts (8 *μ*m pore size; Millipore, Bedford, MA, USA). 600 *μ*l of medium containing 10% FBS was added to the lower chamber. After incubation for 36 h, the cells transferred to the lower surface of the chamber were fixed with methanol, stained with crystal violet, and observed under a microscope (Olympus, Tokyo, Japan).

### 2.7. Western Blot Analysis

Total protein was isolated using RIPA buffer (Beyotime, Shanghai, China). Protein lysates were separated by SDS-polyacrylamide gel electrophoresis and transferred to polyvinylidene difluoride membranes (Millipore). Then, the membranes were blocked with nonfat milk and incubated overnight with the primary antibodies at 4°C, followed by incubation with the HRP-conjugated secondary antibody for 1 h at room temperature. The protein bands were then visualized using an enhanced chemiluminescence reagent (Millipore), and GAPDH was used as a protein-loading control.

### 2.8. Dual-Luciferase Reporter Assay

The fragment of circ-TLK1 or SOX4 mRNA containing the putative binding sites or mutant sites of miR-138-5p was cloned into the pMIR-REPORT vectors (Promega, Madison, WI, USA). Cells were cultured in 24-well plates in advance and cotransfected with the reporter vectors and miR-138-5p mimics or NC mimics using Lipofectamine 2000. At 48 h posttransfection, the cells were lysed, and their luciferase activities were measured by Dual-Luciferase Reporter Assay System (Promega).

### 2.9. Statistical Analysis

All experimental assays were performed in triplicate, and all data were expressed as mean ± standard deviation (SD). Statistical analyses were performed using the GraphPad Prism 6.0 software (GraphPad Software Inc., San Diego, CA, USA) and SPSS 18.0 software (SPSS Inc., Chicago, IL, USA). The continuous data were compared by Student's *t*-test or one-way ANOVA followed by Tukey's test. The categorical parameters were compared with the chi-square test. The correlations were analyzed by Pearson correlation. *P* < 0.05 was considered to indicate a statistically significant difference.

## 3. Results

### 3.1. circ-TLK1 Expression Is Significantly Increased in HCC

**Figure 1:**
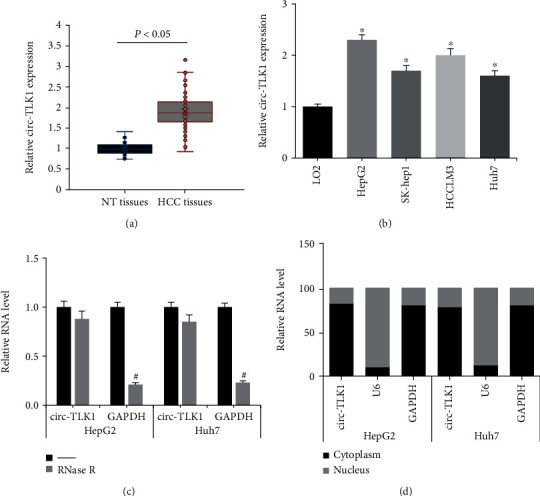
circ-TLK1 expression is significantly increased in HCC. (a) RT-qPCR analysis of circ-TLK1 expression in HCC tissues and adjacent nontumor (NT) tissues. (b) RT-qPCR analysis of circ-TLK1 expression in HCC cell lines and normal LO2 cells. (c) circ-TLK1 and GAPDH expression in HepG2 and Huh7 cells after RNase R digestion. (d) Subcellular localization of circ-TLK1 in HepG2 and Huh7 cells. ^∗^*P* < 0.05 vs. LO2 cells; ^#^*P* < 0.05 vs. RNase R-untreated cells.

We first investigated the expression profile of circ-TLK1 in HCC samples. As demonstrated in Figure 1(a), circ-TLK1 was significantly upregulated in the HCC specimens. According to the mean intratumoral circ-TLK1 expression value, we then divided these patients into high-expression group (*N* = 40) and low-expression group (*N* = 47).  Table 1 suggests that high intratumoral circ-TLK1 expression was closely associated with large tumor size (*P* = 0.030), advanced TNM stage (*P* = 0.018), and vascular invasion (*P* = 0.005) of HCC patients. Besides, circ-TLK1 expression was also markedly increased in four HCC cell lines (HepG2, SK-hep1, HCCLM3, Huh7), compared with normal LO2 cells (Figure 1(b)). Moreover, circ-TLK1 in HepG2 and Huh7 cells was resistant to the digestion of RNase R (Figure 1(c)), confirming the circular property of circ-TLK1. We then determined the subcellular localization of circ-TLK1 in HCC cells and observed that circ-TLK1 was mostly distributed in the cytoplasm of HepG2 and Huh7 cells (Figure 1(d)).

### 3.2. circ-TLK1 Knockdown Inhibits Malignant Behaviors of HCC Cells

**Figure 2:**
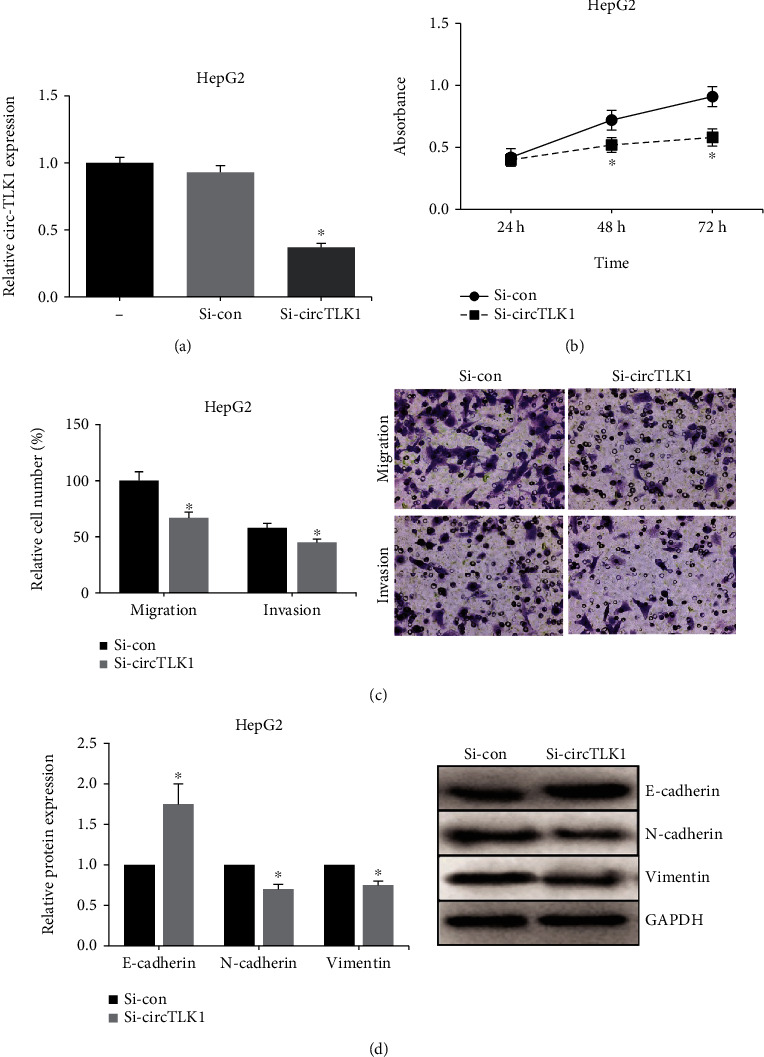
circ-TLK1 knockdown inhibits malignant behaviors of HCC cells. (a) RT-qPCR analysis of circ-TLK1 expression in HepG2 cells after transfection with si-circTLK1. (b) MTT assay of HepG2 cell proliferation after circ-TLK1 knockdown. (c) Transwell assay of HepG2 cell migration and invasion after circ-TLK1 knockdown. (d) Western blot analysis of E-cadherin, N-cadherin, and Vimentin expression in HepG2 cells after circ-TLK1 knockdown. ^∗^*P* < 0.05 vs. si-con-transfected cells.

**Figure 3:**
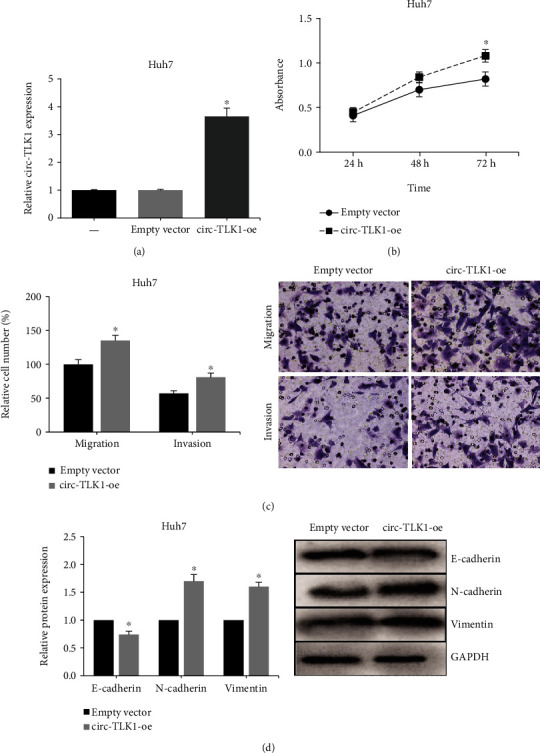
circ-TLK1 overexpression promotes malignant behaviors of HCC cells. (a) RT-qPCR analysis of circ-TLK1 expression in Huh7 cells after transfection with circ-TLK1 overexpression plasmid. (b) MTT assay of Huh7 cell proliferation after circ-TLK1 overexpression. (c) Transwell assay of Huh7 cell migration and invasion after circ-TLK1 overexpression. (d) Western blot analysis of E-cadherin, N-cadherin, and Vimentin expression in Huh7 cells after circ-TLK1 overexpression. ^∗^*P* < 0.05 vs. empty vector-transfected cells.

Given the clinical significance of circ-TLK1 in HCC, we then carried out gain- and loss-of-function assays to investigate the regulatory role of circ-TLK1 in HCC cells. We noted that circ-TLK1 expression in HepG2 cells was obviously decreased after transfection with si-circTLK1 (Figure 2(a)), while circ-TLK1 was overexpressed in Huh7 cells (Figure 3(a)). MTT assay showed that knockdown of circ-TLK1 remarkably inhibited the proliferation of HepG2 cells (Figure 2(b)), while the proliferation of Huh7 cells was accelerated by circ-TLK1 overexpression (Figure 3(b)). Moreover, as shown in Figure 2(c), the migration and invasion of HepG2 cells were also notably suppressed by circ-TLK1 knockdown, while circ-TLK1 overexpression enhanced these abilities of Huh7 cells (Figure 3(c)). Western blot analysis further showed that circ-TLK1 knockdown caused an increased expression of E-cadherin, as well as decreased expressions of N-cadherin and Vimentin in HepG2 cells (Figure 2(d)), while opposite results were observed in Huh7 cells with circ-TLK1 overexpression (Figure 3(d)).

### 3.3. circ-TLK1 Negatively Regulates miR-138-5p in HCC via Target Binding

**Figure 4:**
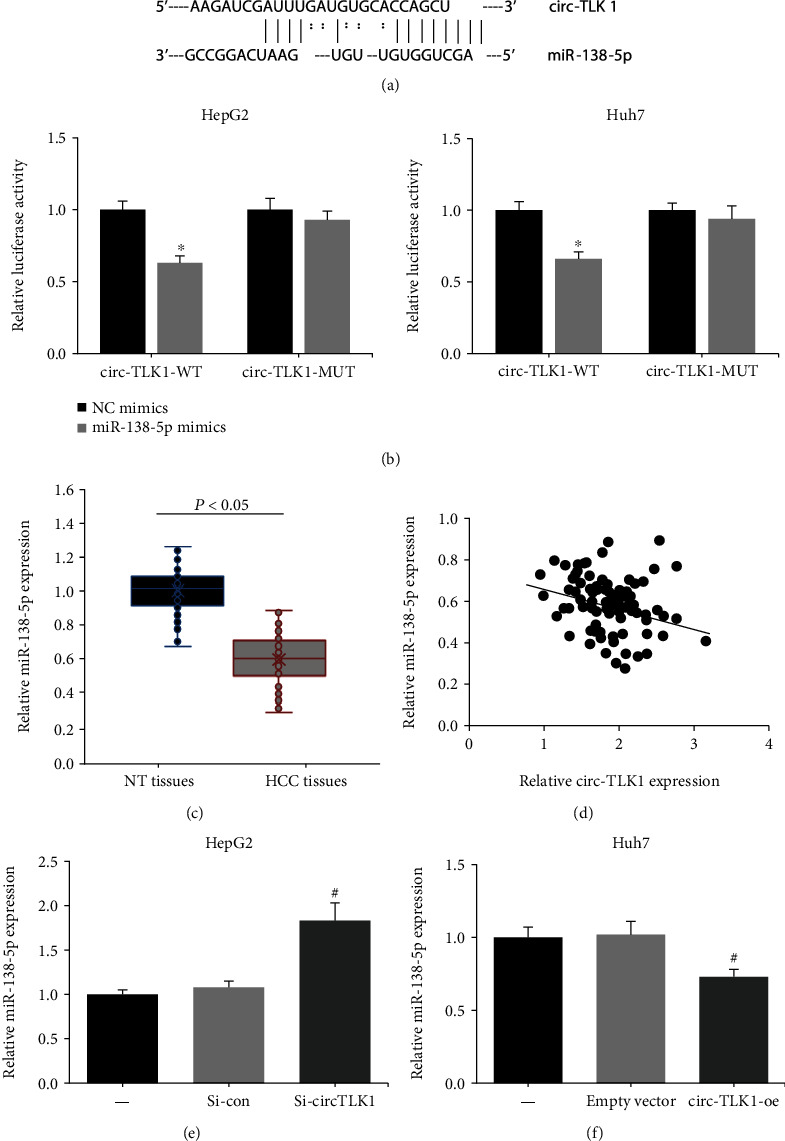
circ-TLK1 negatively regulates miR-138-5p in HCC via target binding. (a) The predicted binding site of miR-138-5p within circ-TLK1 fragment. (b) The luciferase activity of circ-TLK1-WT/MUT in HepG2 and Huh7 cells. (c) RT-qPCR analysis of miR-138-5p expression in HCC tissues and adjacent NT tissues. (d) The correlation between circ-TLK1 and miR-138-5p expression in HCC tissues. (e) RT-qPCR analysis of miR-138-5p expression in HepG2 cells after circ-TLK1 knockdown. (f) RT-qPCR analysis of miR-138-5p expression in Huh7 cells after circ-TLK1 overexpression. ^∗^*P* < 0.05 vs. NC mimics-transfected cells; ^#^*P* < 0.05 vs. si-con/empty vector-transfected cells.

We further explored whether circ-TLK1 could directly bind to miRNAs in HCC. The online software StarBase 3.0 predicted that circ-TLK1 contains the potential complementary binding sites to miR-138-5p (Figure 4(a)). Dual-luciferase reporter assay showed that the luciferase activity of circ-TLK1-WT was remarkably decreased by miR-138-5p mimics in HepG2 and Huh7 cells, while the luciferase activity of circ-TLK1-MUT was not obviously affected (Figure 4(b)). In addition, a lower expression level of miR-138-5p was identified in HCC tissues than in normal tissues (Figure 4(c)), and its expression was negatively correlated with circ-TLK1 in HCC tissues (Figure 4(d)). Moreover, miR-138-5p expression was notably increased in HepG2 cells after circ-TLK1 knockdown (Figure 4(e)), while circ-TLK1 overexpression led to a reduction of miR-138-5p expression in Huh7 cells (Figure 4(f)).

### 3.4. miR-138-5p Inhibition Blocks the Effects of Circ-TLK1 Knockdown on HCC Cells

**Figure 5:**
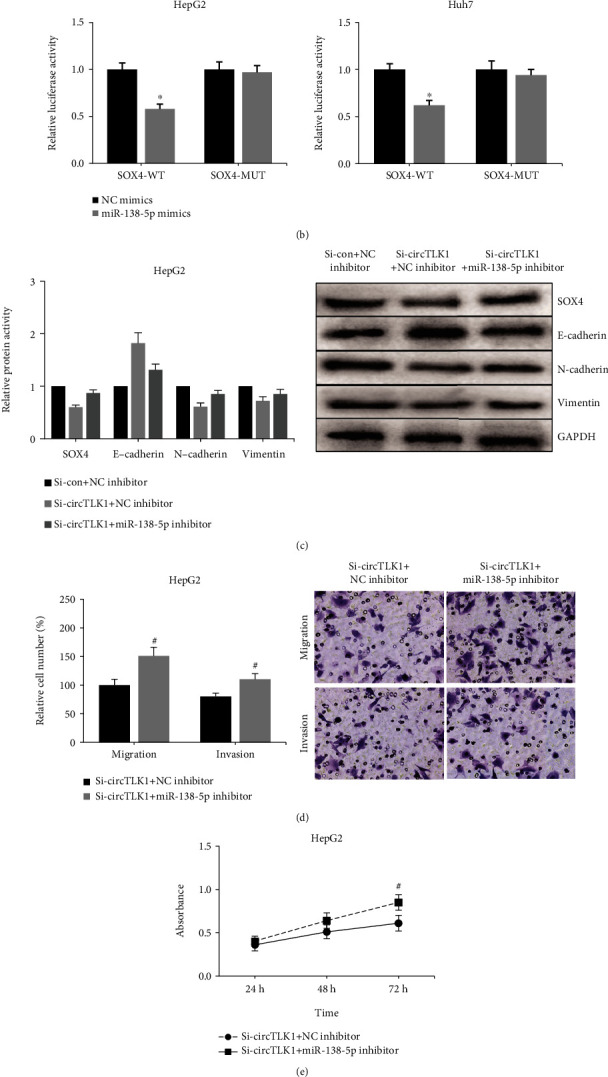
miR-138-5p inhibition blocks the effects of circ-TLK1 knockdown on HCC cells. (a) The predicted binding site of miR-138-5p within SOX4 mRNA fragment. (b) The luciferase activity of SOX4-WT/MUT in HepG2 and Huh7 cells. (c) Western blot analysis of SOX4, E-cadherin, N-cadherin, and Vimentin in HepG2 cell proliferation after miR-138-5p inhibition. (d) Transwell assay of HepG2 cell migration and invasion after miR-138-5p inhibition. (e) MTT assay of HepG2 cell proliferation after miR-138-5p inhibition. ^∗^*P* < 0.05 vs. NC mimics-transfected cells; ^#^*P* < 0.05 vs. NC inhibitor-transfected cells.

Through the TargetScan software, we further found that miR-138-5p could bind to the 3′UTR of SOX4 mRNA (Figure 5(a)). Dual-luciferase reporter assay showed that miR-138-5p mimics markedly reduced the luciferase activity of SOX4-WT in HepG2 and Huh7 cells, while the luciferase activity of SOX4-MUT was not obviously affected (Figure 5(b)). Western blot analysis further showed that miR-138-5p inhibition increased SOX4 protein expression and enhanced the EMT in HepG2 cells with circ-TLK1 knockdown (Figure 5(c)). Moreover, as shown in Figure 5(d), miR-138-5p inhibition obviously abated the inhibitory effects of circ-TLK1 knockdown on the migration and invasion of HepG2 cells. MTT assay also indicated that the restrained proliferation of HepG2 cells with circ-TLK1 knockdown was largely rescued by miR-138-5p inhibition (Figure 5(e)).

## 4. Discussion

circRNAs were first thought to be the products of splicing errors, but in recent years, their roles in carcinogenesis and cancer progression have attracted more and more attention [10, 11]. The validated number of circRNAs involved in HCC continues to increase. They may function as promising diagnostic biomarkers and ideal therapeutic targets for HCC patients [12].

To our knowledge, this is the first report to illustrate the regulatory functions of circ-TLK1 in HCC. Our study disclosed that circ-TLK1 was highly expressed in human HCC samples, and its high expression was closely associated with poor clinicopathological variables of HCC patients. Functional experiments further confirmed that circ-TLK1 knockdown inhibited, while circ-TLK1 overexpression promoted the malignant behaviors of HCC cells, suggesting that circ-TLK1 might act as an oncogene rather than a tumor suppressor in HCC. EMT is a complex biological process that plays a critical role in tumor metastasis [13]. The functional roles of circRNAs during EMT have been widely reported [14], and this study also showed that the EMT of HCC cells could be suppressed by circ-TLK1 knockdown.

circRNAs elicit their biological functions through a plethora of diverse molecular mechanisms. circRNAs have extremely abundant binding sites for microRNAs (miRNAs), and emerging pieces of evidence have suggested that circRNAs can function as miRNA sponges in a ceRNA mechanism to block the inhibitory effects of miRNAs on their target genes [15, 16]. In renal cell carcinoma, circ-TLK1 exerts its oncogenic functions by sponging miR-136-5p [7]. As known, miR-138-5p serves as a tumor suppressor in many malignancies, and a previous article also reported that miR-138-5p restrains the aggressive phenotypes of HCC cells [17]. Here, we selected miR-138-5p as a candidate, and a direct binding correlation between circ-TLK1 and miR-138-5p was identified in HCC. The expression and functions of miR-138-5p in HCC could be negatively regulated by circ-TLK1.

The regulatory roles of miRNAs mainly depend on their target gene(s) [18]. In view of the above results, we then explored the target for miR-138-5p in HCC. SOX4 is frequently overexpressed in over 20 types of malignancies, and it is also widely considered as a master regulator of EMT [19, 20]. SOX4 overexpression may contribute to early recurrence of HCC [21]. This study confirmed that miR-138-5p could directly target SOX4 in HCC. Based on rescue assays, we further noted that miR-138-5p inhibition caused the accumulation of SOX4 protein, thus blocking the effects of circ-TLK1 knockdown on the malignant behaviors of HCC cells.

In summary, the present research, for the first time, revealed that circ-TLK1 is highly expressed in HCC samples and functions as an oncogene in HCC progression partly through competitively binding to miR-138-5p and then relieving the inhibitory effects on SOX4. Our findings may offer a novel therapeutic target, circ-TLK1, to broaden the options for HCC therapy.

## Figures and Tables

**Table 1:** The relationship between circ-TLK1 expression and clinicopathological variables of 87 HCC patients.

Variables	Total number (*N* = 87)	circ-TLK1 expression		*P* value
		Low (*N* = 47)	High (*N* = 40)	
Age (years)				0.354
≤55	46	27	19	
>55	41	20	21	
Gender				0.359
Male	52	26	26	
Female	35	21	14	
Liver cirrhosis				0.112
Yes	60	29	31	
No	27	18	9	
Tumor size (cm)				0.030
<5	50	32	18	
≥5	37	15	22	
TNM stage				0.018
I-II	53	34	19	
III-IV	34	13	21	
Tumor differentiation				0.152
Well-moderate	61	36	25	
Poor	26	11	15	
Vascular invasion				0.005
Yes	30	10	20	
No	57	37	20	

## Data Availability

The data used to support the findings of this study are available from the corresponding author upon request.
